# Supplementary material for: Cancer Stem Cell Markers in Rhabdomyosarcoma in Children
Source: Diagnostics (Basel). 2022 Aug 4;12(8):1895. doi: 10.3390/diagnostics12081895 (PMC9406733; doi:10.3390/diagnostics12081895)
Supplement: Supplementary file 1 [file diagnostics-12-01895-s001.zip › diagnostics-1808002-supplementary.pdf]

Suppl. Table S1. Comparisons of characteristics of T1 and T2 patients.

Variable		T1	T2	P (Fisher test)
**Gender**	female	6	13	1.0
	male	9	21	
**Histologic subtype**	ARMS	5	14	0.7536
	ERMS	10	20	
**Age**	median (IQR)	5.1 (2.6, 8.0)	4.4 (2.3, 9.3)	0.4099[b]

a Fisher's exact test, b Mann-Whitney U test

Suppl. Table S2. Comparisons of characteristics of TNM 1+2+3 and TNM 4 patients.

Variable		TNM 1+2+3	TNM 4	p (Fisher test)
**Gender**	female	13	6	0.7671[a]
	male	19	11	
**Histologic subtype**	ARMS	8	11	0.0127[a]
	ERMS	24	6	
**Age**	median (IQR)	3.9 (2.2, 6.5)	8.3 (3.4, 15.5)	0.0169[b]

a Fisher's exact test, b Mann-Whitney U test

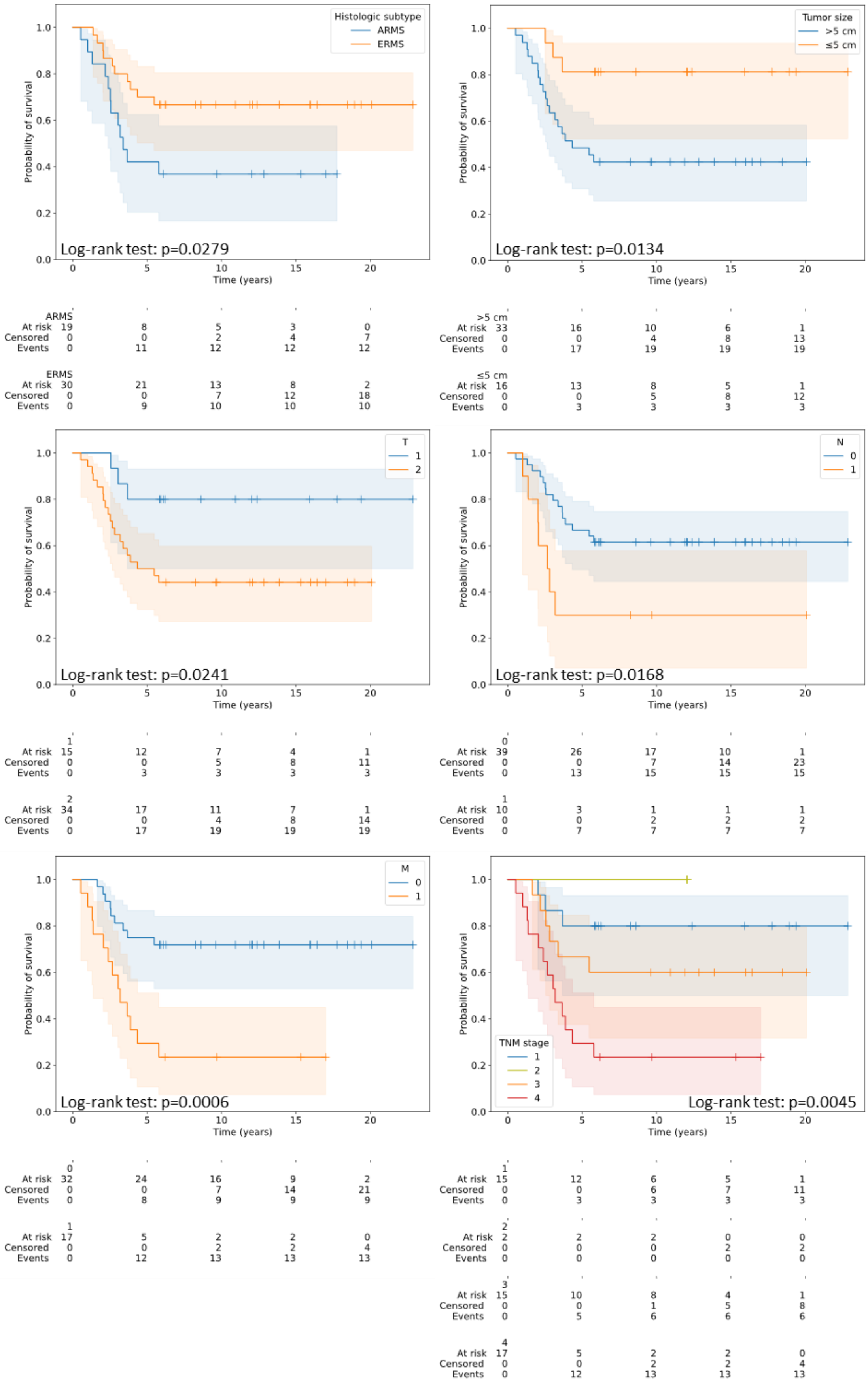

Suppl. Figure S1. Kaplan-Meier curves for significant prognostic predictors.

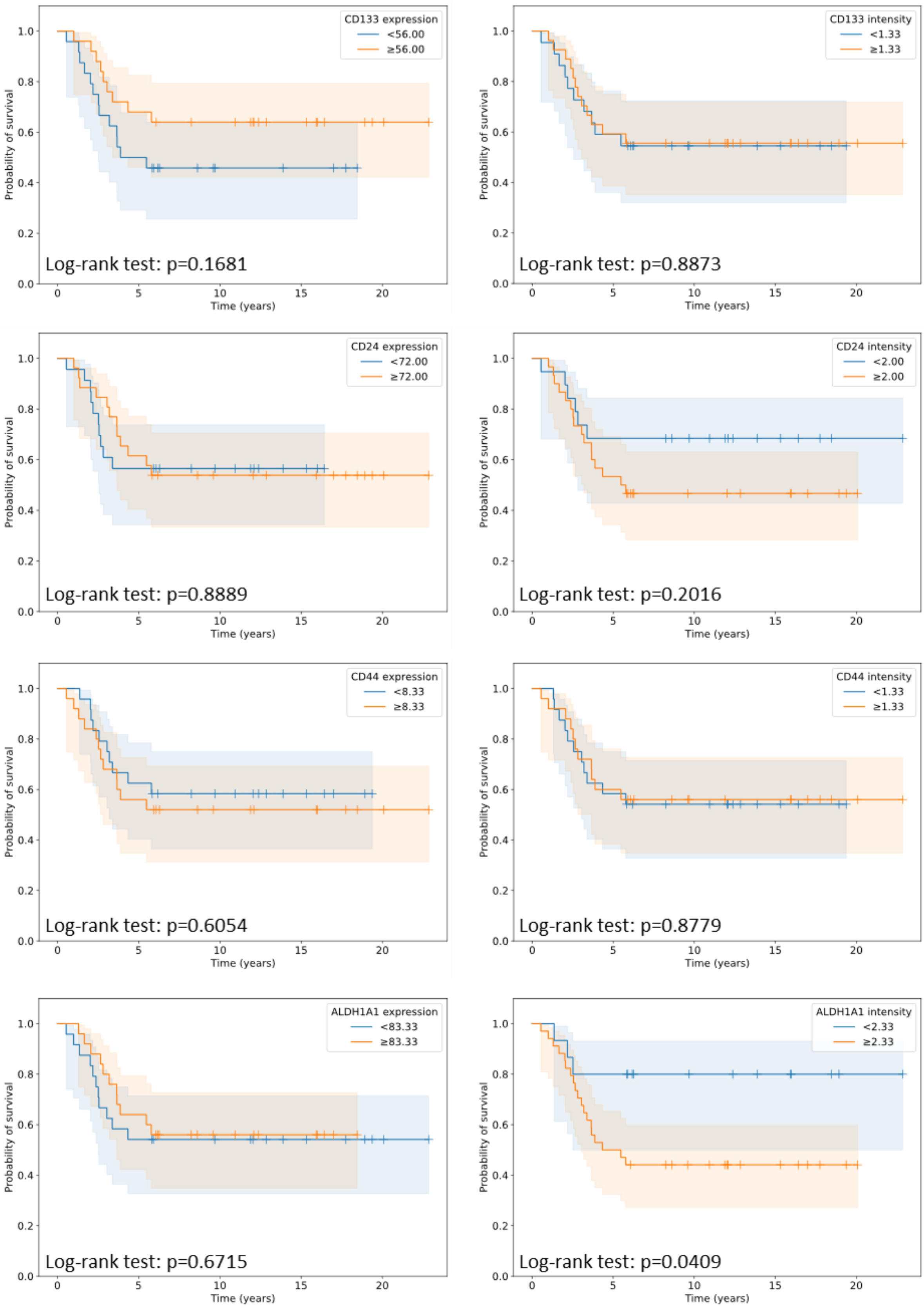

Suppl. Figure S2. Kaplan-Meier curves for CSC markers intensity and expression with cut-off set at median.
